# Anthropometric, Lifestyle and Biomarker Assessment of Japanese Non-professional Ultra-marathon Runners

**DOI:** 10.2188/jea.14.161

**Published:** 2005-03-18

**Authors:** Shinkan Tokudome, Kiyonori Kuriki, Norihiro Yamada, Hiromitsu Ichikawa, Machiko Miyata, Kiyoshi Shibata, Hideki Hoshino, Shinji Tsuge, Mizuho Tokudome, Chiho Goto, Yuko Tokudome, Masaaki Kobayashi, Hideyuki Goto, Sadao Suzuki, Yoshihiro Okamoto, Masato Ikeda, Yuzo Sato

**Affiliations:** 1 Department of Health Promotion and Preventive Medicine, Nagoya City University Graduate School of Medical Sciences.; 2 Division of Cancer Epidemiology and Prevention, Aichi Cancer Center Research Institute.; 3 Kitasato University of Medical Technology.; 4 Kasugai City Health Care Center.; 5 Aichi Bunkyo Women’s College.; 6 Department of Preventive Nutraceutical Sciences, Nagoya City University Graduate School of Pharmaceutical Sciences.; 7 Yokohama Rehabilitation Center.; 8 Nagoya Bunri University.; 9 Department of Bone and Orthopaedics, Nagoya City University Graduate School of Medical Sciences.; 10 Institute of Industrial Ecological Sciences, University of Occupational and Environmental Health.; 11 Department of Health Science, Faculty of Psychological and Physical Sciences, Aichi Gakuin University.

**Keywords:** biomarker measures, health indices, lifestyle-related diseases, physical activity, non-professional ultra-marathon runners

## Abstract

BACKGROUND: Anthropometric characteristics, lifestyle, and baseline biological markers of Japanese non-professional ultra-marathon runners have not been fully assessed.

METHODS: We evaluated anthropometric characteristics, lifestyle, and baseline biological markers of 180 Japanese amateur ultra-marathon runners (144 males [mean age: 50.5±9.4 (standard deviation) years] and 36 females [48.9±6.9]), and compared them with those of participants in a community heath check-up program and with the figures in the literature. We furthermore evaluated baseline blood indices according to monthly running distance with analysis of variance adjusted for age, body mass index, smoking and alcohol drinking habits.

RESULTS: The ultra-marathon runners demonstrated more favorable values for body mass index and bone density, and the proportion of smoking, and undertaking physical activity (for both sexes), eating breakfast (for males), and having daily bowel movements (for females), while greater proportion of alcohol drinking habit (for both sexes), than the comparison group. Average monthly running distances and standard deviations (km) were 257.2±128.9 for males and 209.0±86.2 for females. Male runners possessed beneficial markers, including lowered triglyceride and elevated high-density lipoprotein cholesterol, and their values showed hockey-stick (or inverse hockey-stick) patterns depending on their monthly running distance. Some subjects running more than 300 km/month exhibited signs of an over-reaching/training syndrome, including somewhat lowered hemoglobin, ferritin and white blood cell count, and elevated creatine kinase and lactate dehydrogenase.

CONCLUSIONS: Together with a desirable lifestyle, Japanese non-professional ultra-marathon runners with vigorous exercise habit demonstrated a preferable health status according to biological indices.

There are advantages and disadvantages to physical activity, exercise, and sports. Advantages include elevated bowel motility,^1^^,^^2^ modification of lipid metabolism and amelioration of insulin resistance and glucose intolerance,^3^^-^^5^ improvement of cardiovascular parameters, and prevention of obesity.^6^^,^^7^ Decreased serum concentrations of arachidonic acid and prostaglandin E_2_, reduced generation of radical oxygen species,^8^^-^^15^ enhancing oxygen radicals absorbance capacity and immune surveillance,^16^^-^^19^ including an increased natural killer cell activity, and diminishing cancer risk^1^^,^^2^^,^^20^ may all be achieved. Thus, appropriate physical activity in the long-term may decrease mortality from lifestyle-related diseases, prolong active life expectancy, alleviate mental stress, support mental health and self-efficacy, and finally, enhance quality of life.^6^^,^^7^^,^^21^^-^^23^

On the other hand, disadvantages include damage in the hematopoietic system, skeletal or muscular injuries, oxidative stress/damage, cardiac arrest, arrhythmia, and sudden death.^2^^,^^6^^,^^7^^,^^9^^,^^11^^,^^13^^-^^18^ As is well known, moreover, there exists an over-reaching/training syndrome, and research is needed to clarify the type, intensity, duration, and frequency of physical activity/exercise/sports favorable to our health.

Here, we studied anthropometric characteristics, lifestyle, and baseline biomarker measures among non-professional but vigorously-trained runners entering an ultra-distance race, and compared them with those of people receiving an annual health check-up program and with reference values in the literature. We also assessed baseline blood indices according to their monthly running distance.

## METHODS

### Ultra-marathon race

The ultra-marathon race is not a competitive one. It is nicknamed “*Maranic*” (*mara*thon and *pic*nic), and the tenth race was held in Gifu Prefecture, Japan, during July 27-28, 2002. The midsummer weather was partly cloudy, very hot and sultry. According to the meteorological authority, the temperature was approximately 35°C, and the relative humidity was about 55% at noon on both days. The race covered 130 km of distance running and mountaineering over two days. On the first day, at 11 a.m., the participants started a full-length marathon race to be completed within 6 hr and 30 min. On the second morning, at 3:30 a.m., they resumed the race to run approximately 90 km, including climbing up to a mountain lake approximately 1,100 m high, then returning to the starting point within 15 hr and 30 min.

### Subjects and methods

Six weeks prior to the race, we asked 325 ultra-marathon runners entering the *Maranic* race to enroll in our study. Of these, 202 runners agreed to participate in the project. We received written informed consent from them for completing a questionnaire survey, measuring anthropometric characteristics and bone density, sampling of blood, urine, and saliva, and analyzing genetic polymorphisms, including human 8-oxoguanine DNA glycosylase 1 (*h*-OGG1), aldehyde dehydrogenase 2 (ALDH2), peroxisome proliferators-activated receptor gamma (PPAR *γ*), leptin, angiotensin converting enzyme (ACE), *β*-adrenergic receptor, and CD36 genes. The protocol was approved by the institutional review board of the Nagoya City University Graduate School of Medical Sciences and by the chairman and organizing committee of the race.

We administered our questionnaire to 202 runners by mail and obtained information on anthropometric characteristics and lifestyle, including sex, age (date of birth), height, and dietary, smoking and alcohol drinking habits. We checked unfilled items on the race day along with securing information on smoking, alcohol drinking and supplements taken during the race.

For external comparison, the values of anthropometric characteristics and lifestyle of the participants in a community health check-up program in 2002, except for calcaneal bone density, body temperature, and resting pulse rate, were utilized. We received written informed consent from these participants, and the protocol was approved by the institutional review board of the Nagoya City University Graduate School of Medical Sciences. For calcaneal bone density, body temperature, and resting pulse rate, we used the figures reported in the literature for comparison.^24^^-^^26^

Energy intake was assessed by the short food frequency questionnaire (FFQ).^27^ A regression equation was applied, adopting intake frequency of foods/food groups, average portion size and nutrient concentrations/100 g of foods^28^ as independent variables and energy intake as a dependent variable. Anthropometric measurements and sampling of blood, urine and saliva were performed at the pre- (baseline), mid-, and post-race stages. We measured body weight, body temperature at the tympanum (*Nipro* 43-130, Morishita Jintan, *K.K.*) and blood lactate (*Lactate Pro, LT-1710*, Kyoto Daiichi Kagaku Co., Ltd.). Calcaneal bone density (Speed of Sound) (Stiffness [%]) was gauged ultrasonographically once on three measurement occasions (*A-1000 Express*, GE LUNAR).

We analyzed urine for protein, glucose, occult blood, urobilin, urobilinogen, and pH at the site (*Urisys 2400*, Sysmex K.K.). Baseline serum parameters, including total protein, blood urea nitrogen (BUN), uric acid, aspartate aminotransferase (AST), alanine aminotransferase (ALT), gamma-glutamyltransferase (GTT), lactate dehydrogenase (LDH), creatine kinase (CK), creatinine, total cholesterol, high-density lipoprotein cholesterol (HDL-C), total bilirubin, triglyceride, free fatty acid (*Hitachi 7600*, Hitachi K.K.), myoglobin (radioimmunoassay), lipid peroxide (enzyme method), white blood cells (WBCs), red blood cells (RBCs), hemoglobin (Hb), hematocrit, mean corpuscular volume, mean corpuscular hemoglobin, mean corpuscular hemoglobin concentration, platelets (*XE2100*, Sysmex K.K.), ferritin (chemical immunoluminiscence), HbA_1c_ (HPLC analysis), and serum electrolytes, including sodium (Na), potassium (K), and chlorine (Cl) (*Hitachi 7600*, Hitachi K.K.), were assayed.^29^ Resting pulse in bed in the morning was surveyed by mail after the race.

### Statistical analysis

Anthropometric characteristics and lifestyle values, including bone density,^24^ body temperature at the tympanum,^25^ and resting pulse,^26^ were age-adjusted, adopting the reference population or the study subjects in the literature as standard. The means ±95% confidence interval were computed, and contrasted with those of the participants in a community health check-up program in 2002 or with reference values in the literature. Baseline blood and urine biomarkers were compared with reference values.^29^ Full-length and ultra-marathon completion rates, time and blood indices among males were collated according to monthly running distance (km/month) (≤100, 101-200, 201-300, and 301+) with analysis of variance adjusted for age, body mass index (BMI [kg/m^2^]), smoking, and alcohol drinking.^30^ Tukey’s post hoc multiple t-test was performed to examine differences in the least square means, and the linear trends were statistically verified. The p values smaller than 0.05 were considered statistically significant.

## RESULTS

### Anthropometric measures, and major lifestyle characteristics

Of our participants, 187 runners actually attended the race, and anthropometric measures were taken together with sampling of biomaterials. Seven participants were excluded from the analysis: three with uncompleted questionnaires, and two who were late for the pre-race examination. One finally declined pre- and mid-race blood sampling, and one was excluded due to abnormal liver function. The remaining 180 subjects (144 males and 36 females) were included in this study.

Mean ages were 50.5 ± 9.4 (± standard deviation) years for males and 48.9 ± 6.9 for females, respectively. The differences of means between the groups were obvious: that is, the values for BMI, body temperature (°C), and resting pulse rate were smaller, while those for calcaneal bone density (Stiffness [%] ) (for both sexes) were greater (Table 1). The percentages of smoking and enjoying physical activity (for both sexes), eating breakfast (for males), and having daily bowel movements (for females) were more favorable than for the comparison group. However, the proportion of alcohol drinking habit (for both sexes) was greater in runners than the general public.

**Table 1.** Comparison of anthropometric characteristics and lifestyle between Japanese non-professional ultra-marathon runners and people receiving an annual community health check-up program and other reference people.

Item	males				females			
	
	ultra-marathon runners* (n=144)		reference values^†^		ultra-marathon runners (n=36)		reference values	
Body mass index (kg/m^2^)	22.2	(21.7-22.8)	23.3	(23.1-23.4)	21.2	(20.3-22.1)	22.5	(22.4-22.7)
Eating breakfast (%)	96.5	(94.2-98.9)	90.0	(88.5-91.6)	93.9	(89.5-98.4)	92.4	(91.2-93.6)
Energy intake (kcal)	2,302	(1,936-2,668)	2,117	(2,095-2,139)	1,732	(1,428-2,037)	1,956	(1,942-1,970)
Smoking habit (%)	7.3	(1.7-12.9)	31.1	(28.6-33.6)	1.0	(0-2.4)	6.6	(5.5-7.7)
Alcohol drinking habit (%)	78.6	(70.4-86.7)	58.5	(55.9-61.2)	48.3	(24.1-72.5)	18.7	(16.9-20.4)
Undergoing physical activity (%)	100		40.2	(37.7-42.8)	100		42.1	(39.9-44.3)
Sleep duration (hours)	6.8	(6.6-7.1)	7.0	(6.9-7.0)	6.4	(5.9-6.8)	6.6	(6.6-6.7)
Having daily bowel movements (%)	91.4	(86.0-96.8)	88.9	(87.3-90.6)	96.5	(92.0-100)	70.5	(68.4-72.5)

Calcaneal bone density (Stiffness [%])^‡^	101.2	(97.6-104.7)	85.9	(83.9-87.9)	93.7	(84.6-102.8)	73.5	(72.4-74.6)
Body temperature at the tympanum (°C)^§^	36.2	(36.1-36.4)	36.9	(36.9-36.9)	36.3	(35.9-36.6)	36.9	(36.9-36.9)
Resting pulse (bpm)^||^	53.6	(52.5-54.8)	65.9	(64.2-67.5)	54.7	(52.5-56.9)	66.5	(64.7-68.3)

* : Age-adjusted means ± 95% confidence intervals adopting corresponding reference populations or study subjects in the literature as standard.

† : Values of people receiving annual health check-up program among 1,346 males and 2,043 females, except for calcaneal bone density, body temperature, and resting pulse rate.

‡ : For comparison, values of calcaneal bone density were cited from the reference No. 24.

§ : For comparison, values of body temperature were cited from the reference No. 25.

|| : For comparison, values of resting pulse were cited from the reference No. 26.

### Blood analysis

Average figures for all blood measures were located within the ranges of the reference values (Table 2). They were mostly favorable readings in both sexes. However, hematological markers, such as Hb, ferritin, and WBCs, shifted to be lower than the standard values. On the other hand, damage/repair markers of the musculo-skeletal system, including CK and LDH, tended to be greater than the reference values.

**Table 2.** Comparison of blood indices between Japanese non-professional ultra-marathon runners and reference values.

	males						females					
	
	Ultra-marathon runners (n=144) mean ± standard deviation			Reference values*			Ultra-marathon runners (n=36) mean ± standard deviation			Reference values		
Total protein (g/dL)	7.2	±	0.4	6.7	-	8.3	7.1	±	0.4	6.7	-	8.3
Blood urine nitrogen (BUN, mg/dL)	18	±	4	6	-	20	17	±	4	6	-	20
Uric acid (mg/dL)	5.8	±	1.3	3.7	-	7.6	4.1	±	0.8	2.5	-	5.4

Aspartate aminotransferase (AST, IU/L)	25	±	10	10	-	40	22	±	9	10	-	40
Alanine aminotransferase (ALT, IU/L)	27	±	13	5	-	40	21	±	13	5	-	40
Gamma-glutamyltransferase (GTT, IU/L)	44	±	36		≤	70	21	±	8		≤	30
Lactate dehydrogenase (LDH, IU/L)	198	±	35	115	-	245	197	±	29	115	-	245
Creatine kinase (CK, IU/L)	183	±	139	57	-	197	150	±	91	32	-	180
Creatinine (mg/dL)	0.64	±	0.14	0.61	-	1.04	0.48	±	0.09	0.47	-	0.79
Myoglobin (ng/mL)	43	±	15		≤	60	30	±	9		≤	60

Total cholesterol (mg/dL)	206	±	34	150	-	219	218	±	34	150	-	219
High-density lipoprotein cholesterol (HDL-C, mg/dL)	62	±	15	41	-	86	69	±	14	41	-	96
Total bilirubin (mg/dL)	0.4	±	0.2	0.2	-	1.0	0.4	±	0.2	0.2	-	1.0
Triglyceride (mg/dL)	121	±	66	50	-	149	93	±	35	50	-	149
Free fatty acid (mEq/L)	0.36	±	0.16	0.14	-	0.85	0.35	±	0.17	0.14	-	0.85
Lipid peroxide (nmol/mL)	2.9	±	0.7	1.8	-	4.7	2.6	±	0.6	1.8	-	4.7

White blood cell count (WBCs, /*μ*L)	5,553	±	1,188	3,900	-	9,800	5,197	±	1,281	3,500	-	9,100
Red blood cell count (RBCs, 10^4^/*μ*L)	457	±	38	427	-	570	412	±	30	376	-	500
Hemoglobin (Hb, g/dL)	14.3	±	1.1	13.5	-	17.6	12.7	±	1.1	11.3	-	15.2
Hematocrit (%)	42.9	±	3.2	39.8	-	51.8	39.1	±	2.6	33.4	-	44.9
Mean corpuscular volume (fl)	94.0	±	4.9	82.7	-	101.6	94.8	±	4.2	79.0	-	100.0
Mean corpuscular hemoglobin (pg)	31.2	±	1.7	28.0	-	34.6	30.7	±	1.6	26.3	-	34.3
Mean corpuscular hemoglobin concentration (%)	33.2	±	0.9	31.6	-	36.6	32.4	±	1.0	30.7	-	36.6
Ferritin (ng/mL)	55.4	±	40.3	27	-	320	20.4	±	14.5	3.4	-	89
Platelet count (10^4^/*μ*L)	22.6	±	4.6	13.1	-	36.2	22.2	±	4.0	13.0	-	36.9

Hemoglobin A_1c_ (HbA_1c_, %)	5.1	±	0.4	4.3	-	5.8	4.8	±	0.3	4.3	-	5.8

Sodium (Na, mEq/L)	142	±	2	136	-	147	141	±	2	136	-	147
Potassium (K, mEq/L)	4.1	±	0.6	3.6	-	5.0	4.1	±	0.4	3.6	-	5.0
Chlorine (Cl, mEq/L)	105	±	2	98	-	109	105	±	2	98	-	109

*: Reference values are from the Test Directory 2002.^29^

### Urine analysis

Positive rates for urine glucose of 9.9% for males and 14.6% for females were greater than those in the general people partly because urine was collected on a spot sampling basis (data not shown).

### Completion rates and time according to monthly running distance

Average monthly running distances (km) were 257.2 ± 128.9 (98 - 444) (minimum - maximum) for males and 209.0 ± 86.2 (23 - 222) for females. They mainly selected running, although some concurrently chose swimming, bicycling and other aerobic exercises and occasionally took part in weight/resistance training. Ninety-three percent (167 out of 180 of the study subjects) completed the first day full-length marathon, and 60% (108 out of 180) the 2-day ultra-marathon race. Full-length and ultra-marathon completion rates were positively, while completion time was inversely dependent on their monthly running distance for either sex (data not shown).

### Differences in blood indices according to monthly running distance

The number of male runners by monthly running distance (km/month) of ≤100, 101-200, 201-300, 301+ were 20, 44, 46, and 34, respectively. As a whole, there were no significant discrepancies in anthropometric characteristics, including BMI, body temperature, resting pulse rate and bone density, according to monthly running distance (data not shown). Most blood measurements also showed no remarkable differences in proportion to monthly running distance after adjustment for age, BMI, smoking, and alcohol drinking, either (Table 3). Triglyceride was decreased according to monthly running distance, while HDL-cholesterol was steadily elevated. Some readings, however, revealed hockey-stick (or inverse hockey-stick) patterns, including lowered Hb, ferritin and WBCs, and elevated CK and LDH. The linear trends were statistically/marginally significant except for Hb and triglyceride.

**Table 3.**
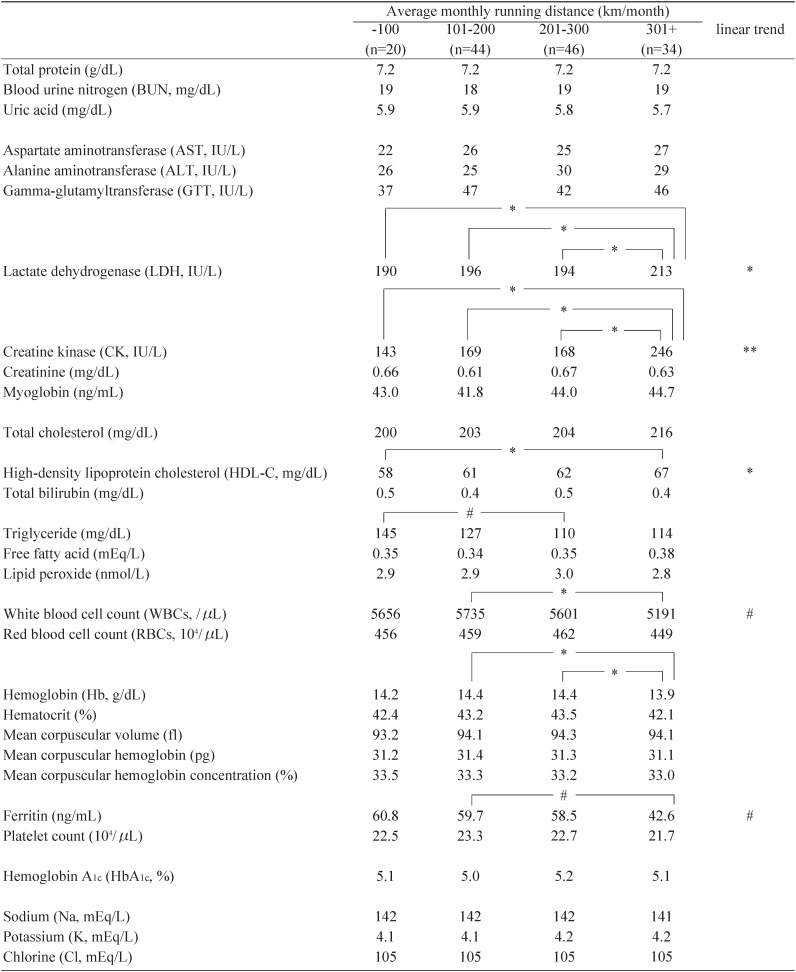
Blood indices according to average monthly running distance adjusted for age, body mass index, smoking and alcohol drinking in Japanese male non-professional ultra-marathon runners.

#: Marginally significant, * p<0.05, ** p<0.01.

## DISCUSSION

Most of the present subjects followed the recommended Seven Heath Practices, including (1) hours of sleep, (2) smoking, (3) body weight, (4) alcohol drinking, (5) physical exercise, (6) eating breakfast, and (7) eating between meals, proposed by Breslow et al.^31^ They showed preferable demographic characteristics and lifestyle, even when compared with health-conscious people receiving an annual health check-up program. Above all, they engaged in vigorous exercise regularly. Most maintained a desirable body weight and bone density,^24^ and smoked less;^32^ however, they experienced greater energy from alcoholic beverages than the general population, at least partly because they regularly expended energy by running. Alcohol consumption, however, up to 30 g net ethanol per day, may not be harmful, provided the subject is not a carrier of hepatitis B/C viruses, or has not hetero/mutant type genetic polymorphisms of ALDH-2.

Most blood measures showed no remarkable variation according to monthly running distance after adjustment for age, BMI, smoking, and alcohol drinking, in line with homeostasis and adaptation.^9^^,^^13^^,^^14^^,^^18^ No significant differences were observed in typical antioxidant molecules of uric acid and bilirubin.^8^^,^^12^ Ferritin levels, however, decreased in proportion to monthly running distance, along with lower body temperature^25^ and resting pulse rates^26^^,^^33^ were noted among the subjects. Taking into account these findings, we are now planning to make pre-, mid- and post-race comparisons of blood, urine and saliva bio-parameters, including serum d-ROM and Mn-SOD, and urinary 8-OHdG and biopyrrins, as markers of reactive oxygen species and oxygen radical absorbance capacity.^8^^-^^15^

Participants running more than 300 km/month exhibited signs of an over-reaching/training syndrome, including lowered Hb, ferritin and WBCs suggesting damage in the hematopoietic system to some degree, and elevated CK and LDH indicating injuries in musculo-skeletal organs. Vigorous exercisers running around 200 km/month, even those running less than 100 km/month, who were insufficiently trained to run an ultra-marathon race, had preferable biomarker indices, implying that such exercises are favorable to health. Namely, triglyceride was decreased and HDL-cholesterol was elevated according to their monthly running distance. Thus, people committed to vigorous exercise probably do not suffer from obesity, high lipidemia/cholesterol,^6^^,^^7^ high blood pressure or high insulin resistance.^3^^-^^5^ Furthermore, they undoubtedly enjoy a low risk of coronary heart disease, cerebrovascular diseases and fat-related cancers, including colon, prostate and breast cancer.^1^^,^^2^^,^^20^

In conclusion, the study subjects were admittedly rather self-selected as being non-professional marathon runners and possessed desirable demographic characteristics and lifestyle, even when compared with health-conscious people receiving an annual health check-up program. Runners committing to vigorous running up to around 200 km/month, but not over-reaching/training, appear to have preferable biomarker indices, suggesting that vigorous aerobic exercise is favorable to health, particularly for sedentary or physically-inactive workers. Further research is warranted to elucidate the type, intensity, duration, and frequency of physical activity/exercise/sports beneficial to promote health, to reduce the risk of lifestyle-related diseases and to enhance the quality of life.
